# The Effect of Cyclosporin A on *Aspergillus niger* and the Possible Mechanisms Involved

**DOI:** 10.3390/foods12030567

**Published:** 2023-01-28

**Authors:** Fengming Li, Zhencheng Lv, Zhijuan Zhong, Lutian Mao, Lee Suan Chua, Liangxiong Xu, Riming Huang

**Affiliations:** 1 School of Life Sciences, Huizhou University, Huizhou 516001, China; 2 College of Food Sciences, South China Agricultural University, Guangzhou 510642, China; 3 Department of Bioprocess and Polymer Engineering, Faculty of Chemical and Energy Engineering, Universiti Teknologi Malaysis, UTM Skudai, Johor Bahru 81310, Malaysia

**Keywords:** cyclosporins A–C, *Aspergillus niger*, antifungal, postharvest grape, transcriptome

## Abstract

*Aspergillus niger* is one of the major pathogenic fungi causing postharvest grape decay. The development of antifungal agents is beneficial to reduce the loss of grapes during storage. The aim of this study was to investigate the antifungal mechanism of cyclosporin A (CsA). It was indicated that the rot development on grapes caused by *A. niger* was almost completely inhibited with CsA in vivo at a concentration of 200 mg/L. The transcriptomic analysis revealed that the expression levels of genes involved in rRNA processing and ribosome biogenesis were down-regulated, whereas those related to *β*-glucosidases and chitinases were up-regulated. The results implied that CsA may disturb rRNA and ribosome formation to obstruct protein synthesis, accelerate chitin and glucan degradation to destruct cell walls, and ultimately reduce postharvest decay caused by *A. niger* in grapes. This study evaluated the potential of CsA as a grape preservative and provided new insights into the mechanisms underlying the molecular response in *A. niger* with the treatment of CsA.

## 1. Introduction

Grapes (*Vitis vinifera* L.) have been cultivated worldwide and consumed by humans for thousands of years [1,2]. As a highly perishable, non-climacteric fruit, grapes are vulnerable to postharvest decay caused by *Aspergillus niger*. This pathogen not only causes significant losses but also produces a large number of mycotoxins, making it one of the main causes of postharvest decay in table grapes [3,4,5]. Currently, sulfur dioxide (SO_2_) fumigation is often used to control *A. niger*, but it has limited effectiveness and can cause bleaching spots on the berries as well as pose health hazards to humans. Therefore, it is important to develop alternative methods to prevent and control postharvest grape decay [6,7,8].

**Figure 1:**
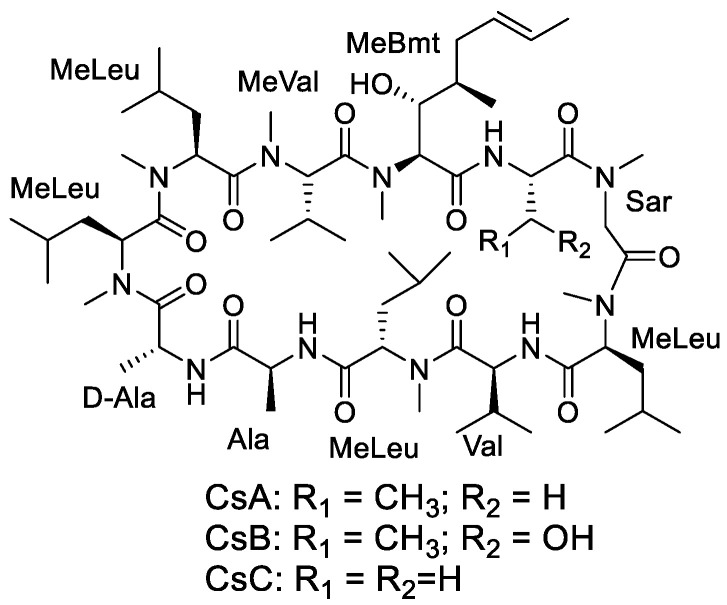
Structures of cyclosporins A–C (CsA–C).

Fungal secondary metabolites are an important ingredient for developing antifungal and antibacterial drugs [9]. The corn fermentation of *Fusarium* sp. HU0298, an endophytic fungus of plants, showed strong antifungal activity against *A. niger*. The subsequent secondary investigation of metabolites led to the isolation of three cyclopeptides: cyclosporins A–C (CsA–C, Figure 1). These compounds were abundant in the metabolites of *Fusarium* sp. HU0298 and showed potent antifungal activities against *Aspergillus* spp. CsA and its antifungal properties have been studied for over 70 years [10,11,12], but the primary mechanism of action, which involves impairing the selective permeability of cytoplasmic membranes and disrupting protein and RNA synthesis in *A. niger*, is not yet fully understood at the transcriptional level [13,14]. In this study, in order to provide further insights into the mechanisms underlying the molecular response in *A. niger* and evaluate the potential of CsA as an antifungal agent, high-dimensional biological data transcriptomics was applied to investigate the effect and the mechanism of CsA on *A. niger*, scanning electron microscopy (SEM) was used to observe the ultrastructural changes in this pathogen after CsA treatment, and the efficacy of CsA against the incidence and severity of black rot disease in postharvest grapes was also studied in vivo.

## 2. Materials and Methods

### 2.1. Producing Fungus and Fermentation

The endophytic fungus *Fusarium* sp. HU0298 was isolated from *Rumex acetosa* collected from the beach near Huanghuashan Village of Nanao County, Guangdong Province, China, in April 2018. It was identified based on its morphological characteristics and ITS sequence data (National Microbiology Data Center number NMDCN0000PR2). The fermentation of this fungus was performed as previously described [15]. The mycelia grown on potato dextrose agar (PDA, Huankai Microbial, Guangdong, China) were prepared, inoculated into two 100 mL Erlenmeyer flasks, each containing 30 mL of potato dextrose broth (PDB, Huankai Microbial, Guangdong, China), and shaken at 28 °C for two days on a rotatory (150 rpm). The culture medium was then transferred into twenty 500 mL culture flasks that each contained 200 mL of PDB at the same incubation conditions. Finally, 5 mL of the culture medium was added into each of five hundred 500 mL culture flasks that each contained 60 mL of pure water and 60 g of corn grains. Fermentation was carried out at 28 °C under stationary conditions for 45 days in the dark.

### 2.2. Preparation of CsA–C

CsA–C were isolated from *Fusarium* sp. HU0298 metabolites and identified using nuclear magnetic resonance (NMR, Quantum-I, 400 MHz, Qone Instruments, Wuhan, China) and electrospray ionization mass spectrometry (ESI-MS, LCMS-8040, Shimadzu, Kyoto, Japan). The obtained solid culture of *Fusarium* sp. HU0298 was extracted three times at room temperature with 95% EtOH. After excluding EtOH, the resulting extract was successively partitioned with petroleum ether and EtOAc. The EtOAc–soluble extract (311.7 g) was separated using a silica gel column and eluted with CH_2_Cl_2_–MeOH mixtures of increasing polarity (100:0 to 80:20) to afford Frs.1–24. Fr. 10 (5.0 g of 24.0 g), obtained via elution of CH_2_Cl_2_–MeOH (90:10), which was then separated with preparative HPLC (LC-16P, Shimadzu, Japan) with 80% aqueous MeOH as the mobile phase to yield CsA (248.6 mg, t*_R_* = 34.7 min, flow rate 4 mL/min), CsB (91.2 mg, t*_R_* = 28.4 min) and CsC (258.0 mg, t*_R_* = 24.2 min). For the ^1^H NMR, ^13^C NMR, (+) ESI-MS, and (−) ESI-MS data, together with the structural determination of CsA-C, see Supplementary Figures S1–S12 [16,17,18].

### 2.3. Fruit Material and Fungal Culture

Grapes (*Vitis vinifera* L. var. Thompson seedless) were harvested in September from a commercial orchard in Shuikou, Huizhou, China. Mature fruits in uniform shape, color, size, and absence of stains or diseases were selected for experiments.

The testing fungus *A. niger* HUSGT008 was isolated from the decayed grapes and stored in our laboratory. The conidia of *A. niger* arthroconidium (1 × 10^6^ CFU/mL) were incubated in the PDA culture at 28 °C for three days and suspended in a sterile physiological saline solution containing 0.5% yeast extract for subsequent use.

### 2.4. Antifungal Activities of CsA–C In Vitro

A paper disk agar diffusion assay was performed to evaluate the antifungal activity of CsA, CsB, and CsC against *A. niger*, *A. japonicas*, *A. tubingensis*, *A. brasiliensis*, and *A. flavus*. Filters (6 mm in diameter) containing 100 μg of the sample were applied to the surface of PDA inoculated with *A. niger* spore suspensions. The inhibition zone diameter was measured after three days of incubation. The mycelial growth inhibition activity of CsA against *A. niger* was also evaluated [19]. Different concentrations of CsA, specifically, 1, 10, 100, 250, 500, 1000, and 10,000 ng/mL, were added to the PDA medium. Methanol and imazalil were used as the negative and positive controls, respectively.

### 2.5. Effect of CsA on the Disease Development in Postharvest Grapes Inoculated with A. niger

**Figure 2:**
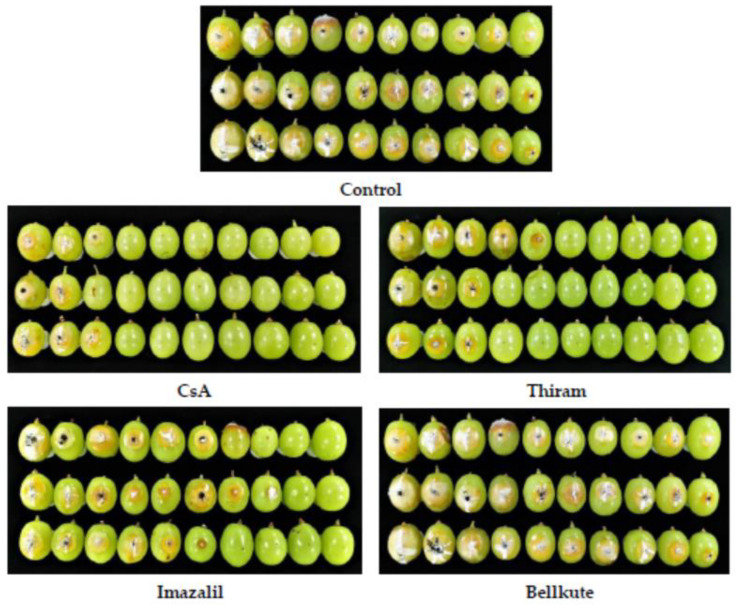
The disease control efficacy of control (0.1% ethanol), cyclosporin A (CsA), thiram, imazalil, and bellkute with concentration at 50 mg/L against *A. niger* on grapes.

Grapes were treated as per Duan et al. [20,21]. The wounded fruits were then treated for 3 min with 50, 100, and 200 mg/L of CsA in 0.1% ethanol. Subsequently, 5 μL of spore suspension was added to the wound with a pipette and incubated at 28 °C. The fruit was infiltrated with 0.1% ethanol without CsA as a negative control, whereas thiram, bellkute, and imazalil were used as positive controls. The experiment was repeated in triplicate, with each treatment consisting of 10 berries. Each row of grape berries in the figure represents a treatment repetition (Figure 2). All treated berries were sealed in plastic containers and stored at 28 °C in the dark for 7 days.

The disease incidence was calculated using the following formula: disease incidence (%) = decay number/10 × 100%.

### 2.6. Scanning Electron Microscopy (SEM) Observation

Fungal mycelia grown on PDB medium for three days with 1 mg/L CsA were observed using SEM [22]. The ultrastructures of the samples were observed using a JSM-6360 LV scanning electron microscope (NEC, Tokyo, Japan).

### 2.7. RNA Extraction and Real-Time Quantitative PCR (RT-qPCR) Analysis

The 3-day-old mycelia of *A. niger* grown in the PDB medium with or without 1 mg/L CsA were collected. The total RNA was extracted using the Hipure Fungal RNA Mini Kit (Magen, Guangzhou, China) and purified with DNase (TaKaRa Bio, Inc., Otsu, Shiga, Japan). DNA-free RNA was reverse-transcribed for first-strand cDNA synthesis. The specific primers designed with Primer Premier 6.0 are shown in Supplementary Table S1. Q-PCR was performed with a 7500 Fast Real-Time PCR System (Applied Biosystems, Foster City, CA, USA). Actin was used as the housekeeping gene to normalize the cDNA content. The formula 2^−ΔΔCT^ was used for calculating the relative expression levels of the target genes [23]. Three independent biological replicates were used in the experiment.

### 2.8. RNA-Seq Analysis

RNA samples from the same treatment in Section 2.7 were used for RNA-Seq analysis. RNA-Seq was performed as previously described [24]. RNA concentration was determined using Qubit, and RNA quality was evaluated with a NanoDrop spectrophotometer. The mRNA was purified, adenylated, ligated, built, and then sequenced on an Illumina Hiseq 4000 platform. Single-read sequencing (expected library size, 150 base pairs; read length, 50 nucleotides) was performed in the present study. The analysis of differentially expressed genes (DEGs) was based on the Poisson distribution method. For the significance of digital gene expression profiles, Padj < 0.05 and |log2 foldchange| ≥ 2 were used as the threshold. RNA-Seq analysis was performed in triplicate.

### 2.9. Statistical Analysis

The data are expressed as mean ± standard deviation. The mean separations were analyzed using Duncan’s multiple range tests, and differences between treatments were determined using SPSS version 25 at the 5% level.

## 3. Results and Discussion

### 3.1. Antifungal Activities of CsA–C against A. niger In Vitro

**Figure 3:**
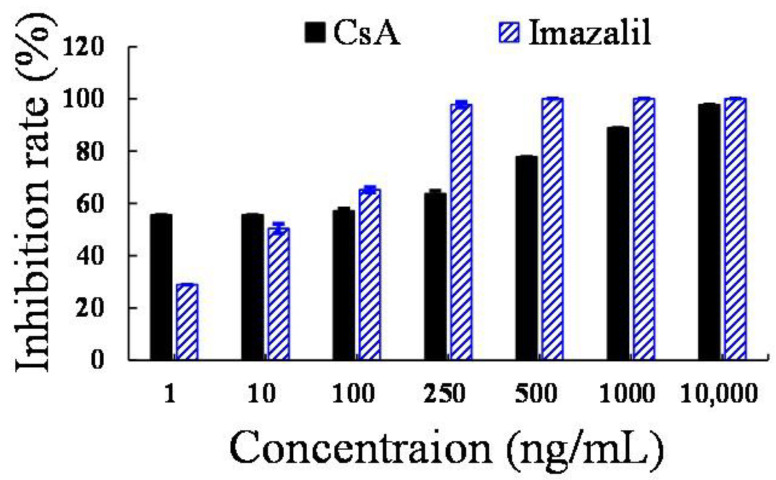
The inhibition rates of cyclosporin A (CsA) and imazalil at different concentrations on the mycelial growth of *A. niger* after three days of incubation.

*Aspergillus* spp. is one of the most common pathogenic fungi in fruits and vegetables. CsA, CsB, and CsC exhibited potent antifungal activity at a dose of 100 μg. The inhibition zone radius of CsA was greater than those of CsB and CsC (Table 1). Moreover, CsA strongly inhibited the mycelial growth of *A. niger* at a concentration of 1 ng/mL (Figure 3). Colony diameter decreased by 55.6% after three days of treatment with 1 ng/mL CsA for, which was significantly higher than the positive control, imazalil (28.9%).

### 3.2. Inhibition Effects of CsA on Disease Development in Artificially Inoculated Grapes

CsA showed strong inhibitory effects on *A. niger* growth in vitro. The control grape berries showed a disease incidence of 100%, whereas the disease incidences of grape berries treated with 50 and 100 mg/L CsA were 26.7 and 23.3%, respectively. CsA almost completely inhibited the rot development on grapes caused by *A. niger* at a concentration of 200 mg/L, with a disease incidence of 6.7% (Table 2). These results showed that CSA significantly inhibited the growth of *A. niger*. Moreover, the coverage area of mycelial growth was significantly larger than the wounded area in control, whereas most grape berries treated with CsA showed minimal browning around the inoculation site (wound) and little mycelial growth (Figure 2). Furthermore, the therapeutic effect of CsA was better than that of the positive controls thiram, bellkute, and imazalil at a concentration of 50 mg/L, with disease incidences of 36.7, 40.0, and 70.0%, respectively.

There are numerous latent diseases in fruits, which usually occur in postharvest storage. The most common postharvest pathogenic fungi reported for grape berries are *B. cinerea*, *A. niger,* and *Penicillium expansum* [25,26]. Thiram, bellkute, and imazalil have been widely used for postharvest preservation of fruits and vegetables in past decades [27,28,29]. CsA showed a significantly stronger effect than bellkute and imazalil at 50 mg/L in vivo. The results demonstrate that CsA effectively inhibits the growth and reproduction of *A. niger* in grapes and may prolong the storage life of grapes.

### 3.3. Morphology and Ultrastructural Alterations of A. niger

**Figure 4:**
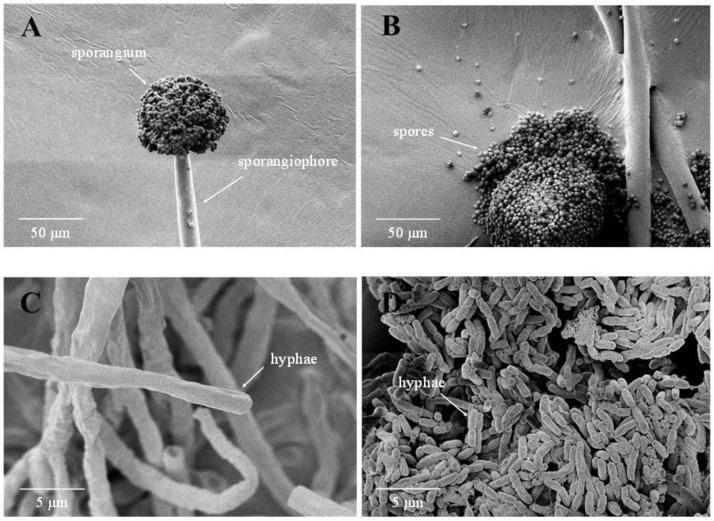
Scanning electron micrographs of *Aspergillus niger* cultured on PDB for 3 days. (**A**–**C**) Control; (**D**) treated with cyclosporin A (CsA) at 1 mg/L.

The hyphae and sporangiophores in the control groups appeared regular, smooth, and normal after three days of cultivation. The spores and sporangium showed a regular and spherical shape with a clear outline (Figure 4A–C). However, treatment with 1 mg/L CsA significantly inhibited the hyphae growth of *A. niger*, resulting in a short and rhabdoid shape, and no sporangia or spores were detected (Figure 4D).

### 3.4. Transcriptomic Analysis of A. niger in Response to CsA

**Figure 5:**
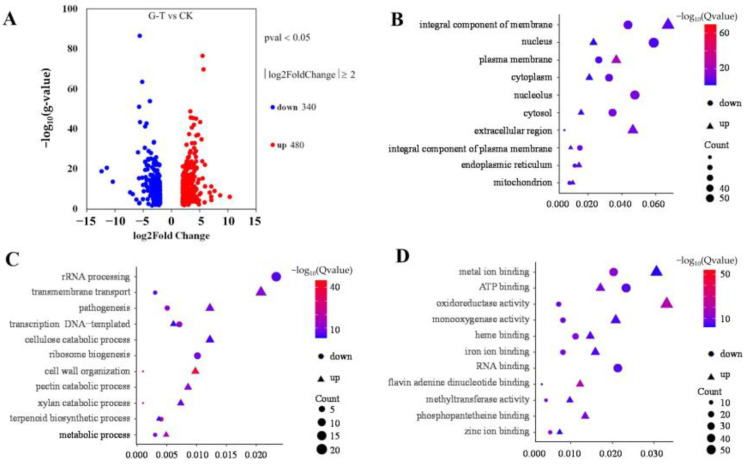
Volcano plots of differentially expressed genes (DEGs) in *Aspergillus niger* (**A**); bubble chart of cell components (**B**), biological processes (**C**), and molecular functions (**D**) of DEGs in GO enrichment analysis.

The transcriptomes of mycelium samples were analyzed to determine the global RNA changes induced by CsA treatment at 1 mg/L. In total, 13,095 genes were detected in both the control and treatment groups. After CsA treatment, 820 DEGs were obtained, with 480 of them up-regulated and 340 down-regulated (Figure 5A). Based on the cell components category, DEGs belonging to “membrane” and “nucleus/nucleolus” were enriched (Figure 5B). GO enrichment analysis was also performed to investigate the biological functions of these DEGs. DEGs associated with “rRNA processing” and “ribosome biogenesis” were enriched and down-regulated (Figure 5B). However, DEGs for cell wall organization and xylan catabolism were up-regulated. According to the recorded molecular functions, DEGs for “metal ion binding,” “ATP binding,” “oxidoreductase activity,” and “RNA binding” were enriched (Figure 5C).

**Figure 6:**
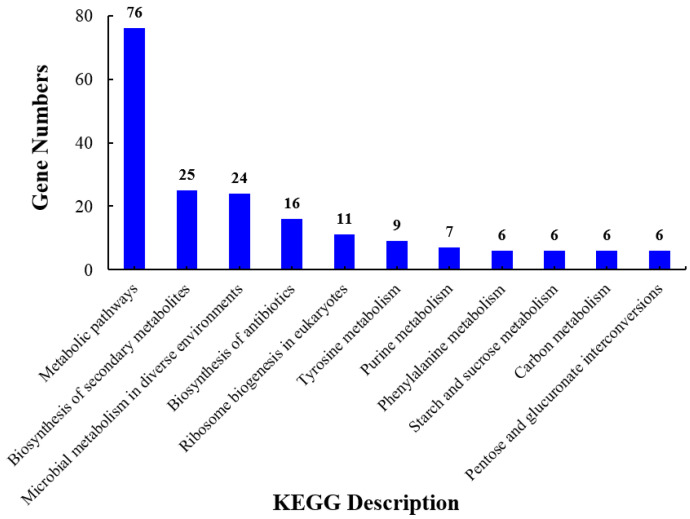
KEGG pathway classification of the top 11 differentially expressed genes.

**Figure 7:**
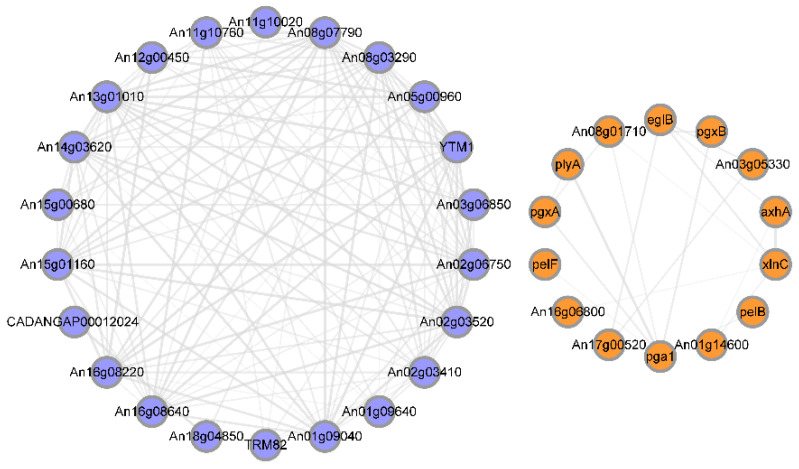
Protein-protein interaction (PPI) network of the 36 representative DEGs. Colored nodes represent query proteins and first shell of interactors. Edges represent protein–protein associations.

Furthermore, the first ten variable pathways from a total of 128 KEGG pathways were used to screen out important genes in the KEGG enrichment pathway. Most of the genes were enriched in metabolic pathways. Ribosome biogenesis in eukaryotes, starch and sucrose metabolism, carbon metabolism, and pentose and glucuronate interconversions were also involved (Figure 6). Based on GO enrichment, KEGG pathways, and the String database, 36 genes were identified as important genes associated with the results of the phenotypic study; their PPI networks and descriptions are shown in Figure 7 and Table S2, respectively. It is hypothesized that the antifungal mechanism of CsA involves rRNA synthesis, ribosome biogenesis, and fungal cell wall disruption.

Ribosomes and rRNA are essential for protein synthesis, which is necessary for biological growth, development, and reproduction. The DEGs of rRNA processing and ribosome biogenesis were down-regulated in the top eleven variable categories of biological processes (Figure 5C). ATP-dependent RNA helicases (An08g07790, An01g09040, An15g01160, and An02g06750) were involved in multiple rRNAs or in pre-rRNA formation. The keys to tRNA processing and maturation were the methyltransferases of structural modification of tRNA (An01g09640, TRM82, An01g00070, and An02g03410). tRNA maturation was closely related to the transport of protein synthesis precursors and products [30]. DNA-directed RNA polymerase III subunits (CADANGAP00012024 and An18g04850) affected DNA transcription into RNA using the four ribonucleoside triphosphates as substrates [31,32]. RNA recognition motif family proteins (An11g10760, An11g10020, An16g08640, CAN33_4095, and CAN33_4035) played an essential role in rRNA processing [33]. The component of the NOP7 complex (YTM1) was required for 25S, and 5.8S rRNA maturation and RNA-3’-phosphate cyclase family protein (An16g08220) were required for 18S rRNA synthesis [34]. The proteins (An05g00960, An12g00450, CAN33_2620, and An03g06850) were involved in rRNA processing and maturing. Furthermore, ribosome biogenesis regulatory proteins (An13g01010, YTM1, An02g03520, CAN33_5400, and An15g00680) were involved in ribosome biogenesis and subunit assembly, especially the formation of the 60S ribosome [35]. Moreover, the nucleolar protein consisted of ribosomal RNA processing proteins (An08g03290 and An14g03620). These results indicate that the down-regulation of rRNA processing and ribosome biogenesis genes may reduce the protein required for normal cell biogenic activities and growth.

It is well known that α-amanitin, a highly toxic cyclopeptide isolated from *Amanita* spp., can inhibit mRNA synthesis by interrupting RNA polymerase II translocation and suppressing nuclear maturation [36]. Rifampicin, an antibiotic for the treatment of nocardiosis and numerous mycobacterial infections, can inhibit protein biosynthesis by blocking RNA transcription in bacteria by binding to the *β*-subunit of the DNA-dependent RNA polymerase [37]. Sazykin et al. suggested that cyclosporine affects the RNA and protein synthesis of *A. niger* [14], but the molecule mechanism was not explained. CsA may inhibit the transcription and translation of RNA polymerase III complex in the current study. The results demonstrated that CsA treatment inhibited DNA transcription, tRNA transport, and the processing and maturation of *5.8S*, *18S,* and *25S* rRNA, as well as the biogenesis of *40S* and *60S* ribosome subunits and assembly in the cell, inhibiting *A. niger* growth as a result. SEM observations revealed that the growth and development of mycelium treated with CsA was inhibited and could not develop completely (Figure 4D).

The cell wall organization is the most significant pathway in the biological process (Figure 5C). The glucosidases (An03g05330, An17g00520, and eglB), xylanases (xlnC and An01g14600), furanosidases (axhA and An08g01710), galacturonases (pgxA, pgxB, pga1, and An11g00390), and Pectin lyases (pelB and pelf) involved in cell wall organization were up-regulated. Moreover, the expression levels of chitinases (CAN33_0014840 and P36362) were significantly up-regulated with the log2 fold changes of 1.8 and 1.2, respectively (Table S2). The results indicated that the antifungal mechanism of CsA was related to the upregulation of cell wall hydrolase expression.

The fungal cell wall can be targeted with antifungal drugs [38]. It was reported that 1, 3-*β*-D-glucan and chitin were the most abundant cell wall polymers of both yeasts and filamentous fungi as well as the important components of the supra-molecular complex in the cell wall [39,40]. *β*-1,3-glucanase and chitinase may play an important role in cell wall remodeling and modification [41]. Dreyfuss et al. reported that CsA blocked chitin synthesis in *Neurospora crassa* [10]. The cell wall organization pathway was the most significant biological process in this study (Figure 5C). Several regulation-related genes in *A. niger* were significantly up-regulated in response to CsA. The *β*-glucosidases (An17g00520, An03g05330, An16g06800, and eglB) with various endoglucanase activities may be involved in 1, 3-*β*-D-glucan degradation [42]. In contrast, chitinases (CAN33_0014840 and P36362, with the log2 fold changes of 1.8 and 1.2, respectively) had endo-hydrolysis of N-acetyl-*β*-D-glucosaminide (1→4)-*β*-linkages in chitin and chitodextrins (Table S2) [43,44]. Furthermore, the phenomenon of hyphae splitting into particles was observed (Figure 4D). High expression of *β*-glucosidases and chitinases enhanced cell wall hydrolase activity that augmented cell wall destruction, thereby inhibiting the growth of *A. niger* after CsA treatment.

**Figure 8:**
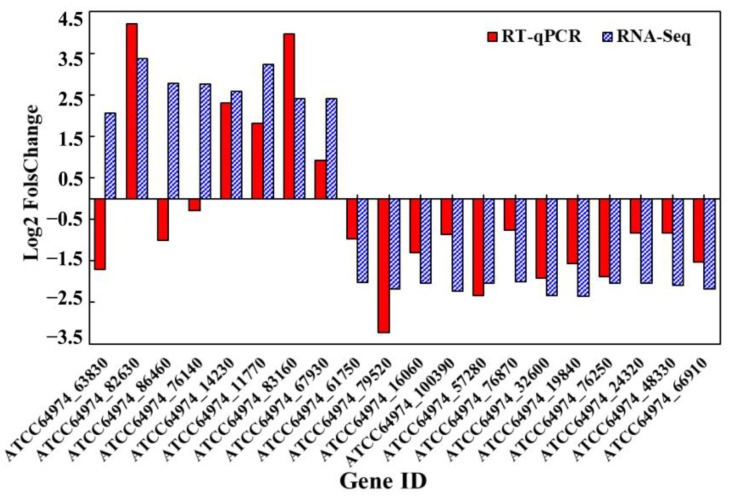
Validation of DEGs by comparing qRT-PCR analysis and RNA-Seq analysis.

The expression levels of some DEGs from the PPI network were analyzed using RT-qPCR analysis to confirm the effect of CsA treatment on *A. niger*. The expression patterns of 17/20 genes were consistent with that of the transcriptome (Figure 8). The results indicated that the transcriptomic analysis was stable and reliable. Transcriptomic analysis revealed that the antifungal mechanism of CsA was related to rRNA processing, ribosome biogenesis, and cell wall hydrolysis. Moreover, CsA was shown to induce leakage of low-molecular weight intracellular components by impairing the selective permeability of cytoplasmic membranes in *A. niger* [13]. In conclusion, CsA can significantly inhibit the mycelial growth of fungi by interrupting protein synthesis from the “inside” and by destroying the integrity and permeability of the cell membrane and cell wall from the “outside.”

## 4. Conclusions

The antifungal mechanism of CsA was systematically investigated for the first time by using transcriptomic and ultrastructural observations. The results revealed that CsA treatment can inhibit the growth of *A. niger*, disrupt normal morphology, and impede protein synthesis by disturbing rRNA and ribosome formation. Additionally, CsA accelerates the degradation of chitin and glucan in the cell wall, ultimately leading to its destruction. These findings provide a new understanding of the inhibitory mechanisms of CsA and can serve as a reference for evaluating its potential as a preservative for grapes. To ensure the safe utilization of CsA, future studies should focus on assessing its toxicity during consumption as well as determining optimal dosage and application methods. Only with this information can CsA be considered for widespread use.

## Figures and Tables

**Table 1:** Inhibition zone radius (mm) of cyclosporins A–C (CsA–C) against *A. niger*.

Compound	CsA	CsB	CsC
*A. niger*	8.0 ± 0.0 ^a^	7.0 ± 0.0 ^b^	4.2 ± 0.2 ^c^

Different letters within a column of the same Aspergillus strain represent significant differences according to the least significant difference (LSD) test (*p* < 0.05).

**Table 2:** The disease incidences of cyclosporin A (CsA) against *A. niger* on grapes.

Treatment	Concentration (mg/L)	Disease Incidence (%) ^†^
control	0	100.0 ± 0.0 a
CsA	50	26.7 ± 4.7 c
	100	23.3 ± 4.7 c
	200	6.7 ± 4.7 d
Thiram	50	36.7 ± 9.4 c
	100	43.3 ± 9.4 c
	200	23.3 ± 4.7 c
Bellkute	50	40.0 ± 8.2 c
	100	23.3 ± 4.7 c
	200	0.0 ± 0.0 d
Imazalil	50	70.0 ± 8.2 b
	100	26.7 ± 9.4 c
	200	10.0 ± 0.0 d

^†^ Mean ± standard deviations indicated with letters are significantly different according to the least significant difference (LSD) test (*n* = triplicates; *p* < 0.05).

## Data Availability

Data are contained within the article.

## Supplementary Materials

The following supporting information can be downloaded at: https://www.mdpi.com/article/10.3390/foods12030567/s1, Table S1: Primers used for real-time PCR; Table S2: Representative DEGs in different comparisons between CsA treatment and control; Figures S1–S12: NMR spectra and ESI-MS for CsA–C.
