# Supplementary material for: The Effect of Cyclosporin A on Aspergillus niger and the Possible Mechanisms Involved
Source: Foods. 2023 Jan 28;12(3):567. doi: 10.3390/foods12030567 (PMC9913951; doi:10.3390/foods12030567)
Supplement: Supplementary file 1 [file foods-12-00567-s001.zip › foods-2103602-supplementary.pdf]

The effect of cyclosporin A on *Aspergillus niger* and the possible mechanisms involved

Fengming Li [1,2,†], Zhencheng Lv [1,†], Zhijuan Zhong [1], Lutian Mao [1], Lee Suan Chua [3], Liangxiong Xu [1,*] and Riming Huang [2,*]

[1] School of Life Sciences, Huizhou University, Huizhou 516001, China
[2] College of Food Sciences, South China Agricultural University, Guangzhou 510642, China
[3] Department of Bioprocess and Polymer Engineering, Faculty of Chemical and Energy Engineering, Universiti Teknologi Malaysia, UTM Skudai, Johor Bahru 81310, Malaysia

* Correspondence: xlx048@hzu.edu.cn (L.X.); huangriming@scau.edu.cn (R.H.)
† These authors contributed equally to this work.

Supplementary Materials

Contents：

Table S1. Primers used for real-time PCR. 3

Table S2. Representative DEGs in CsA treatment and control. 5

Structural identification of cyclosporin A–C. 7

Figure S1. ^{1}H NMR of cyclosporin A.. 9

Figure S2. ^{13}C NMR of cyclosporin A.. 9

Figure S3. (+) ESI-MS of cyclosporin A.. 10

Figure S4. (−) ESI-MS of cyclosporin A.. 10

Figure S5. ^{1}H NMR of cyclosporin B.. 11

Figure S6. ^{13}C NMR of cyclosporin B.. 11

Figure S7. (+) ESI-MS of cyclosporin B.. 12

Figure S8. (−) ESI-MS of cyclosporin B.. 12

Figure S9. ^{1}H NMR of cyclosporin C.. 13

Figure S10. ^{13}C NMR of cyclosporin C.. 13

Figure S11. (+) ESI-MS of cyclosporin C.. 14

Figure S12. (−) ESI-MS of cyclosporin C.. 14

Table S1. Primers used for real-time PCR.

Gene name	Primers (5′-3′)	Description
Actin	F: GGTTTCCTCAAGGTCGGATATG<br>R: CCCATCTCTTCACGATACCATTC	House-keeping gene
ATCC64974_63830	F: TCCTTGCTGACAACGGCGATGC<br>R: TTGGCGTAGGTGACGGTGAGGT	Pectate lyase A
ATCC64974_82630	F: CGCCGTGAACAACCTCTTCCACAA<br>R: GGTGAGCCGCAGCAATGGTCTT	Pectin lyase B
ATCC64974_86460	F: GTAGCCCTTGTCGGAAACGCAGAT<br>R: CACACGGACGCATCGCCAAAGA	Pectinesterase
ATCC64974_76140	F: CCTCCTTCCCTTGACGCTCTTCCT<br>R: CTTCAACAACAACCGACCGCCATG	Exopolygalacturonase B
ATCC64974_14230	F: TCTCCGACTCCACTGTCAGCAACT<br>R: GAGCCGTCACCGCAGAGAATGTAG	Endopolygalacturonase I
ATCC64974_11770	F: GACAGCGACGACGGAACCTACAAG<br>R: AGTCTCAACGGTGATGGTGGCAGA	Endo-1,4-β-xylanase
ATCC64974_83160	F: GCACTTTCACCTACCGCACCTCTC<br>R: GTACACTTGGACCGCCTCGAACAA	α-L-arabinofuranosidase
ATCC64974_67930	F: ACCGAGTGGCTGAAGACCAACAAC<br>R: GTAGCAGCAGCAACGGAGACAGAG	Cellulase A
ATCC64974_61750	F: AACGGTGGTGCTGGTGTCTACAAC<br>R: TCGTCGGACTCGTAGTAGCCATCG	Nucleolar GTP-binding protein 1
ATCC64974_79520	F: TGGCGAGGAGGGCGAGAAGATT<br>R: AGCCTCAGGAGCGGTCTCATCAA	Nucleolar GTP-binding protein 2
ATCC64974_16060	F: CAGAAGCAGCAGAAGCGGACCAA<br>R: ACGGGAACACGAGCCTCCTTGA	ATP-dependent RNA helicase has1
ATCC64974_100390	F: GATGCGGACGGAACGGAGAAGAAG<br>R: GTGCCCTTGCCCTTGAACTTGAGA	ATP-dependent RNA helicase dbp4

Gene name	Primers (5′-3′)	Description
ATCC64974_76870	F: GCGATGAGGATGAGTCTGGCGATG<br>R: GTCACCGAACTGGCTGAAGTAGGC	RNA recognition motif protein
ATCC64974_32600	F: CCTTGCAGATGCGAGTTCGACCAA<br>R: CCTATGGACCGAGCCGTTGATGTG	tRNA dimethylallyltransferase
ATCC64974_19840	F: GCCACACAGCATTCATCAGCAAGC<br>R: CCGTCAGATTCCAGCGTGAAGCAT	tRNA methyltransferase
ATCC64974_76250	F: CGAAGCACGCAAGCACCATGAAG<br>R: GCGGCTCGACGATCCTTGATTCTT	rRNA-processing protein
ATCC64974_24320	F: TTCTCAACCAGCTCCTGACCACCT<br>R: CACCAACTTCTTGCGACGCTCCT	Ribosome biogenesis regulatory protein
ATCC64974_48330	F: TCGTCGTCGCAGGCTGGAAAGA<br>R: GCCATCACTCTCATCGCCGTCATC	rRNA-processing protein
ATCC64974_66910	F: TGCTGGGTCTGATGACGATGGGAT<br>R: ATGGTGCTCTCCTCGGCGATCA	rRNA-processing protein

Table S2. Representative DEGs in different comparisons between CsA treatment and control.

Gene name	Log2 foldchange	Name	Description
ATCC64974_75020	2.148092932	An03g05330	β-glucosidase
ATCC64974_64540	2.042347189	An17g00520	β-glucosidase I
ATCC64974_49560	2.247971421	eglB	Endo-β-1,4-glucanase B
ATCC64974_67930	2.403118414	An16g06800	Cellulase
ATCC64974_83150	3.045727063	xlnC	Endo-1,4-β-xylanase C
ATCC64974_83160	2.414639663	axhA	α-L-arabinofuranosidase
ATCC64974_11770	3.222379082	An01g14600	Endo-1,4-β-xylanase
ATCC64974_104980	2.121349655	An08g01710	α-N-arabinofuranosidase C
ATCC64974_90170	2.537519625	pgxA	Exopolygalacturonase A
ATCC64974_76140	2.762757751	pgxB	Exopolygalacturonase B
ATCC64974_14230	2.583143671	pga1	Endopolygalacturonase I
ATCC64974_87310	2.383571245	An11g00390	Rhamnogalacturonate lyase B
ATCC64974_63830	2.049605059	plyA	Pectate lyase A
ATCC64974_82630	3.375258584	pelB	Pectin lyase B
ATCC64974_27210	2.035303708	pelF	Pectin lyase F
ATCC64974_86460	2.774728183	CAN33_7630	Pectinesterase
ATCC64974_26570	1.75310009	CAN33_0014840	Chitinase
ATCC64974_62990	1.207602158	P36362	Chitinase
ATCC64974_28340	-2.3651205	CADANGAP00012024	RNA Polymerase C
ATCC64974_107890	-2.133346352	An18g04850	DNA-directed RNA polymerase subunit
ATCC64974_100390	-2.228547666	An08g07790	ATP-dependent RNA helicase dbp4
ATCC64974_16060	-2.04063327	An01g09040	ATP-dependent RNA helicase has1
ATCC64974_31800	-2.381563875	An15g01160	ATP-binding RNA helicase Dhr1p
ATCC64974_57280	-2.040507378	An02g06750	ATP-dependent RNA helicase dbp8
ATCC64974_19840	-2.358190465	TRM82	tRNA methyltransferase non-catalytic subunit
ATCC64974_15590	-2.235025771	An01g09640	SAM-binding methyltransferase

Gene name	Log2 foldchange	Name	Description
ATCC64974_23420	-2.302264715	An01g00070	SAM-binding methyltransferase
ATCC64974_61670	-2.240805505	An02g03410	Methyltransferase
ATCC64974_66910	-2.186110032	An16g08220	18S rRNA biogenesis protein RCL1
ATCC64974_38490	-2.058772523	An05g00960	rRNA-processing protein
ATCC64974_24320	-2.03698352	An13g01010	Ribosome biogenesis regulatory protein
ATCC64974_78040	-2.148006262	YTM1	Ribosome biogenesis protein
ATCC64974_61750	-2.013841311	An02g03520	Nucleolar GTP-binding protein 1
ATCC64974_79520	-2.180820626	CAN33_5400	Nucleolar GTP-binding protein 2
ATCC64974_39330	-2.061517517	An12g00450	Ribosomal RNA-processing protein 7
ATCC64974_48330	-2.095671872	CAN33_2620	rRNA-processing protein efg1
ATCC64974_76250	-2.03094042	An03g06850	rRNA-processing protein
ATCC64974_32160	-2.545837203	An15g00680	60S ribosomal subunit assembly/export protein loc1
ATCC64974_103830	-2.420533415	An08g03290	Nucleolar protein Nop52
ATCC64974_3200	-2.275908849	An14g03620	Nucleolar protein 9
ATCC64974_95370	-2.260509495	An11g10760	RNA recognition motif protein NRS1
ATCC64974_94820	-2.093839892	An11g10020	RNA recognition motif protein
ATCC64974_66650	-2.1447343	An16g08640	RNA recognition motif protein
ATCC64974_76870	-2.007029001	CAN33_4095	RNA recognition motif protein gar2
ATCC64974_76770	-2.266362682	CAN33_4035	RNA recognition motif protein

Structural identification of cyclosporin A–C.

Cyclosporine A (CsA): white amorphous solid; ^{1}H NMR ($CDCl_3$, 400 MHz): *1-MeBmt*: δ 5.44 (d, *J* = 5.8 Hz, 2H, H-2), 5.33 (m, 1H, H-6, 7), 3.80 (m, 1H, H-3), 3.49 (s, 3H, NCH_3), 2.37 (m, 1H, H-5), 1.61 (m, 1H, H-4, 5), 1.61 (m, 3H, H-8), 0.70 (d, *J* = 5.8 Hz, 3H, H-9); *2-Abu*: δ 7.92 (d, *J* = 8.9 Hz, 1H, NH), 5.01 (m, 1H, H-2), 1.72 (m, 2H, H-3), 0.89 (m, 3H, H-4); *3-Sar*: δ 4.72 (d, *J* = 14.0 Hz, 1H, H-2), 3.37 (s, 3H, NCH_3), 3.17 (s, 1H, H-2); *4-MeLeu*: δ 5.30 (m, 1H, H-2), 3.10 (s, 3H, NCH_3), 2.22 (m, 1H, H-3), 1.61 (m, 1H, H-3), 1.43 (m, 1H, H-4), 1.02 (m, 3H, H-5), 0.83 (m, 3H, H-6); *5-Val*: δ 7.46 (d, *J* = 8.4 Hz, 1H, NH), 4.65 (m, 1H, H-2), 2.40 (m, 1H, H-3), 1.04 (d, *J* = 9.6 Hz, 3H, H-4), 0.83 (m, 3H, H-5); *6-MeLeu*: δ 4.98 (m, 1H, H-2), 3.23 (s, 3H, NCH_3), 2.22 (m, 2H, H-3), 1.61 (m, 1H, H-4), 0.83 (m, 3H, H-5, 6); *7-Ala*: δ 7.68 (d, *J* = 7.4 Hz, 1H, NH), 4.50 (m, 1H, H-2), 1.34 (s, 3H, H-3); *8-Ala*: δ 7.15 (d, *J* = 7.6 Hz, 1H, NH), 4.81 (m, 1H, H-2), 1.24 (s, 3H, H-3); *9-MeLeu*: δ 5.68 (d, *J* = 4.1 Hz, 1H, H-2), 3.08 (s, 3H, NCH_3), 1.94 (m, 2H, H-3), 1.61 (m, 1H, H-4), 0.83 (m, 3H, H-5, 6); *10-MeLeu*: δ 5.07 (m, 1H, H-2), 2.68 (s, 3H, NCH_3), 1.94 (m, 2H, H-3), 1.61 (m, 1H, H-4), 0.83 (m, 3H, H-5, 6); *11-MeVal*: δ 5.12 (d, *J* = 11.0 Hz, 1H, H-2), 3.70 (s, 3H, NCH_3), 1.94 (m, 2H, H-3), 0.83 (m, 3H, H-4, 5). ^{13}C NMR ($CDCl_3$, 100 MHz): *1-MeBmt*: δ 171.6 (C-1), 129.7 (C-6), 126.3 (C-7), 74.8 (C-3), 58.8 (C-2), 36.0 (C-4, N-C), 35.7 (C-5), 18 (C-8), 16.8 (C-9); *2-Abu*: δ 173.6 (C-1), 48.9 (C-2), 25.4 (C-3), 10.0 (C-4); *3-Sar*: δ 171.2 (C-1), 50.4 (C-2), 34.0 (N-C); *4-MeLeu*: δ 170.5 (C-1), 55.5 (C-2), 37.4 (C-3), 31.6 (N-C), 24.9 (C-4), 21.2 (C-6), 18.8 (C-5); *5-Val*: δ 173.8 (C-1), 48.3 (C-2), 23.8 (C-3), 20.3 (C-5), 18.5 (C-4); *6-MeLeu*: δ 170.4 (C-1), 55.4 (C-2), 39.1 (C-3), 31.4 (N-C), 25.1 (C-4), 23.9 (C-5), 21.9 (C-6); *7-Ala*: δ 171.2 (C-1), 48.7 (C-2), 16.1 (C-3); *8-Ala*: δ 173.7 (C-1), 45.2 (C-2), 18.2 (C-3); *9-MeLeu*: δ 170.1 (C-1), 55.4 (C-2), 39.5 (C-3), 31.2 (N-C), 24.6 (C-4), 23.9 (C-5), 21.9 (C-6); *10-MeLeu*: δ 170.1 (C-1), 57.6 (C-2), 40.7 (C-3), 29.9 (N-C), 24.7 (C-4), 23.9 (C-5), 23.4 (C-6); *11-MeVal*: δ 173.5 (C-1), 58.0 (C-2), 29.8 (N-C), 29.1 (C-3), 23.5 (C-5), 19.9 (C-4). (+)-ESIMS m/z 1224 $[M + Na]^+$; (–)-ESIMS m/z 1200 $[M – H]^-$. Based on these results, the compound was identified as cyclosporine A [16].

Cyclosporine B (CsB): white amorphous solid; ^{1}H NMR ($CDCl_3$, 400 MHz): *1-MeBmt*: δ 5.41 (m, 1H, H-2), 5.34 (m, 1H, H-6, 7), 3.78 (m, 1H, H-3), 3.48 (s, 3H, NCH_3), 2.37 (m, 1H, H-5), 1.61 (m, 1H, H-4, 5), 1.61 (m, 3H, H-8), 0.70 (d, *J* = 5.8 Hz, 3H, H-9); *2-Abu*: δ 8.00 (d, *J* = 10.2 Hz, 1H, NH), 5.08 (m, 1H, H-2), 1.02 (m, 3H, H-3); *3-Sar*: δ 4.73 (m, 1H, H-2), 3.38 (s, 3H, NCH_3); *4-MeLeu*: δ 5.34 (m, 1H, H-2), 3.10 (s, 1H, NCH_3), 2.22 (m, 1H, H-3), 1.61 (m, 1H, H-3), 1.43 (m, 1H, H-4), 1.02 (m, 3H, H-5), 0.83 (m, 3H, H-6); *5-Val*: δ 7.39 (d, *J* = 8.0 Hz, 1H, NH), 4.67 (m, 1H, H-2), 2.40 (m, 1H, H-3), 1.04 (d, *J* = 9.6 Hz, 3H, H-4), 1.02 (m, 3H, H-5); *6-MeLeu*: δ 4.99 (m, 1H, H-2), 3.23 (s, 3H, NCH_3), 2.22 (m, 2H, H-3), 1.61 (m, 1H, H-4), 1.02 (m, 3H, H-5), 0.83 (m, 3H, H-6); *7-Ala*: δ 7.68 (d, *J* = 7.4 Hz, 1H, NH), 4.51 (m, 1H, H-2), 1.34 (s, 3H, H-3); *8-Ala*: δ 7.14 (d, *J* = 8.0 Hz, 1H, NH), 4.82 (m, 1H, H-2), 1.24 (s, 3H, H-3); *9-MeLeu*: δ 5.68 (d, *J* = 6.9 Hz, 1H, H-2), 3.09 (s, 3H, NCH_3), 2.22 (m, 2H, H-3), 1.61 (m, 1H, H-4), 1.02 (m, 3H, H-5), 0.83 (m, 3H, H-6); *10-MeLeu*: δ 5.23 (m, 1H, H-2), 2.68 (s, 3H, NCH_3), 2.22 (m, 2H, H-3), 1.61 (m, 1H, H-4), 1.02 (m, 3H, H-5), 0.83 (m, 3H, H-6); *11-MeVal*: δ 5.13 (m, 1H, H-2), 2.71 (s, 3H, NCH_3), 2.22 (m, 2H, H-3), 1.02 (m, 3H, H-4), 0.83(m, 3H, H-5). ^{13}C NMR ($CDCl_3$, 100 MHz): *1-MeBmt*: δ 171.6 (C-1), 129.6 (C-6), 129.4 (C-7), 74.8 (C-3), 58.6 (C-

2), 36.2 (C-4), 36.1 (N-C), 35.8 (C-5), 18.0 (C-8), 16.6 (C-9); *2-Abu*: δ 173.5 (C-1), 43.1 (C-2), 17.8 (C-3); *3-Sar*: δ 171.6 (C-1), 50.4 (C-2), 33.9 (N-C); *4-MeLeu*: δ 171.0 (C-1), 55.6 (C-2), 37.4 (C-3), 31.6 (N-C), 24.8 (C-4), 22.2 (C-6), 18.9 (C-5); *5-Val*: δ 174.0 (C-1), 48.4 (C-2), 23.8 (C-3), 20.2 (C-5), 18.7 (C-4); *6-MeLeu*: δ 170.1 (C-1), 55.4 (C-2), 39.1 (C-3), 31.3 (N-C), 25 (C-4), 23.9 (C-5), 21.9 (C-6); *7-Ala*: δ 171.3 (C-1), 48.8 (C-2), 16.2 (C-3); *8-Ala*: δ 173.6 (C-1), 45.3 (C-2), 18.3 (C-3); *9-MeLeu*: δ 170.1 (C-1), 55.3 (C-2), 39.4 (C-3), 31.3 (N-C), 24.7 (C-4), 23.8 (C-5), 22.1 (C-6); *10-MeLeu*: δ 169.9 (C-1), 57.6 (C-2), 40.8 (C-3), 29.9 (N-C), 25.5 (C-4), 23.8 (C-5), 23.3 (C-6); *11-MeVal*: δ 173.5 (C-1), 58.0 (C-2), 29.8 (N-C), 29.0 (C-3), 23.5 (C-5), 19.7 (C-4). (+)-ESIMS m/z 1210 $[M + Na]^+$; (–)-ESIMS m/z 1186 $[M – H]^-$. Based on these results, the compound was identified as cyclosporine B [17].

Cyclosporine C (CsC): white amorphous solid; ^{1}H NMR ($CDCl_3$, 400 MHz): *1-MeBmt*: δ 5.33 (m, 1H, H-2, 6, 7), 3.90 (m, 1H, H-3), 3.48 (s, 3H, NCH_3), 2.37 (m, 1H, H-5), 1.61 (m, 1H, H-4, 5), 1.61 (m, 3H, H-8), 0.70 (d, *J* = 5.8 Hz, 3H, H-9); *2-Abu*: δ 8.20 (d, *J* = 10.1 Hz, 1H, NH), 5.10 (m, 1H, H-2), 4.06 (m, 1H, H-3); *3-Sar*: δ 4.74 (m, 1H, H-2), 3.33 (s, 3H, NCH_3); *4-MeLeu*: δ 5.10 (m, 1H, H-2), 3.10 (s, 3H, NCH_3), 2.22 (m, 1H, H-3), 1.61 (m, 1H, H-3), 1.43 (m, 1H, H-4), 1.02 (m, 3H, H-5), 0.83 (m, 3H, H-6); *5-Val*: δ 7.21 (d, *J* = 7.1 Hz, 1H, NH), 4.74 (m, 1H, H-2,), 2.40 (m, 1H, H-3), 1.04 (d, *J* = 9.6 Hz, 3H, H-4), 0.83 (m, 3H, H-5); *6-MeLeu*: δ 4.87 (m, 1H, H-2), 3.17 (s, 3H, NCH_3), 2.22 (m, 2H, H-3), 1.61 (m, 1H, H-4), 1.02 (m, 3H, H-5), 0.83 (m, 3H, H-6); *7-Ala*: δ 8.02 (d, *J* = 7.1 Hz, 1H, NH), 4.41 (m, 1H, H-2), 1.34 (s, 3H, H-3); *8-Ala*: δ 7.11 (d, *J* = 9.5 Hz, 1H, NH), 4.74 (m, 1H, H-2), 1.24 (s, 3H, H-3); *9-MeLeu*: δ 5.65 (d, *J* = 7.6 Hz, 1H, H-2), 3.03 (s, 3H, NCH_3), 2.22 (m, 2H, H-3), 1.61 (m, 1H, H-4), 1.02 (m, 3H, H-5), 0.83 (m, 3H, H-6); *10-MeLeu*: δ 5.10 (m, 1H, H-2), 2.66 (s, 3H, NCH_3), 2.22 (m, 2H, H-3), 1.61 (m, 1H, H-4), 1.02 (m, 3H, H-5), 0.83 (m, 3H, H-6); *11-MeVal*: δ 5.22 (m, 1H, H-2), 2.71 (s, 3H, NCH_3), 2.22 (m, 2H, H-3), 1.02 (m, 3H, H-4), 0.83 (m, 3H, H-5). ^{13}C NMR ($CDCl_3$, 100 MHz): *1-MeBmt*: δ 171.4 (C-1), 126.5 (C-7), 129.4 (C-6), 74.3 (C-3), 58.8 (C-2), 35.9 (C-4), 35.3 (N-C), 35.0 (C-5), 17.9 (C-8), 16.5 (C-9); *2-Abu*: δ 173.2 (C-1), 67.3 (C-3), 50.0 (C-2), 28.9 (C-4); *3-Sar*: δ 171.1 (C-1), 51.5 (C-2), 33.5 (N-C); *4-MeLeu*: δ 170.9 (C-1), 55.4 (C-2), 37.5 (C-3), 31.8 (N-C), 24.9 (C-4), 21.4 (C-6), 18.9 (C-5); *5-Val*: δ 173.9 (C-1), 48.2 (C-2), 23.7 (C-3), 20.1 (C-5), 18.7 (C-4); *6-MeLeu*: δ 170.6 (C-1), 54.6 (C-2), 39.2 (C-3), 31.4 (N-C), 25.1 (C-4), 23.9 (C-5), 21.7 (C-6); *7-Ala*: δ 170.9 (C-1), 48.9 (C-2), 16.3 (C-3); *8-Ala*: δ 173.5 (C-1), 45.3 (C-2), 18.1 (C-3); *9-MeLeu*: δ 170.0 (C-1), 54.6 (C-2), 39.2 (C-3), 31.2 (N-C), 24.7 (C-4), 23.7 (C-5), 21.7 (C-6); *10-MeLeu*: δ 170.2 (C-1), 57.3 (C-2), 40.8 (C-3), 29.9 (N-C), 24.7 (C-4), 23.7 (C-5), 23.2 (C-6); *11-MeVal*: δ 173.2 (C-1), 58.1 (C-2), 29.9 (N-C), 29.8 (C-3), 23.4 (C-5), 19.1 (C-4). (+)-ESIMS m/z 1240 $[M + Na]^+$; (–)-ESIMS m/z 1216 $[M – H]^-$, 1252 $[M + Cl]^-$. Based on these results, the compound was identified as cyclosporine C [18].

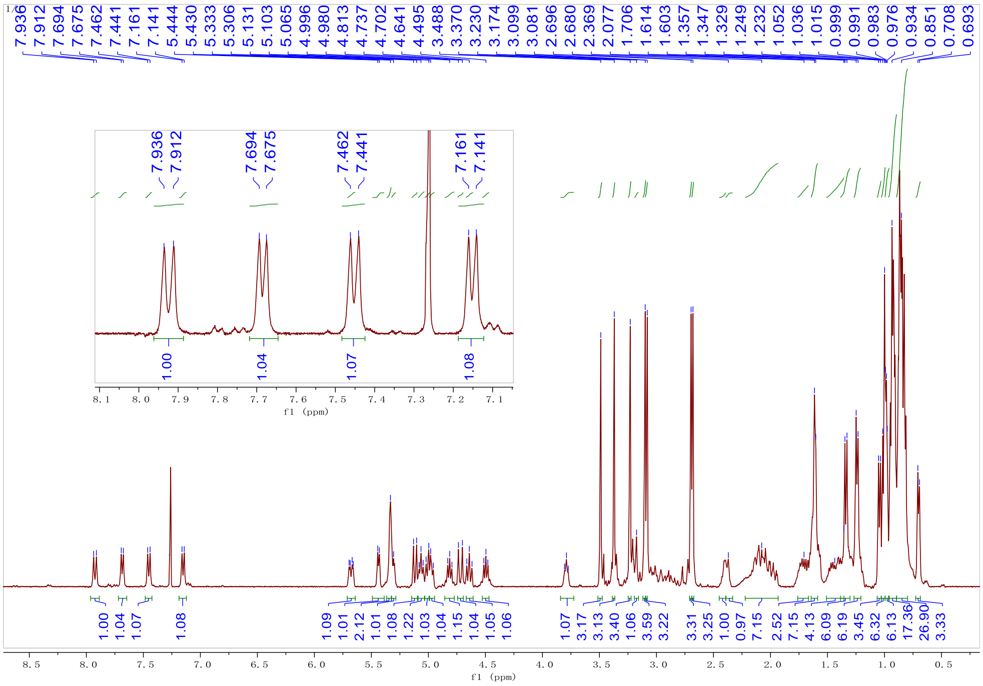

Figure S1. ^{1}H NMR of cyclosporin A (CsA).

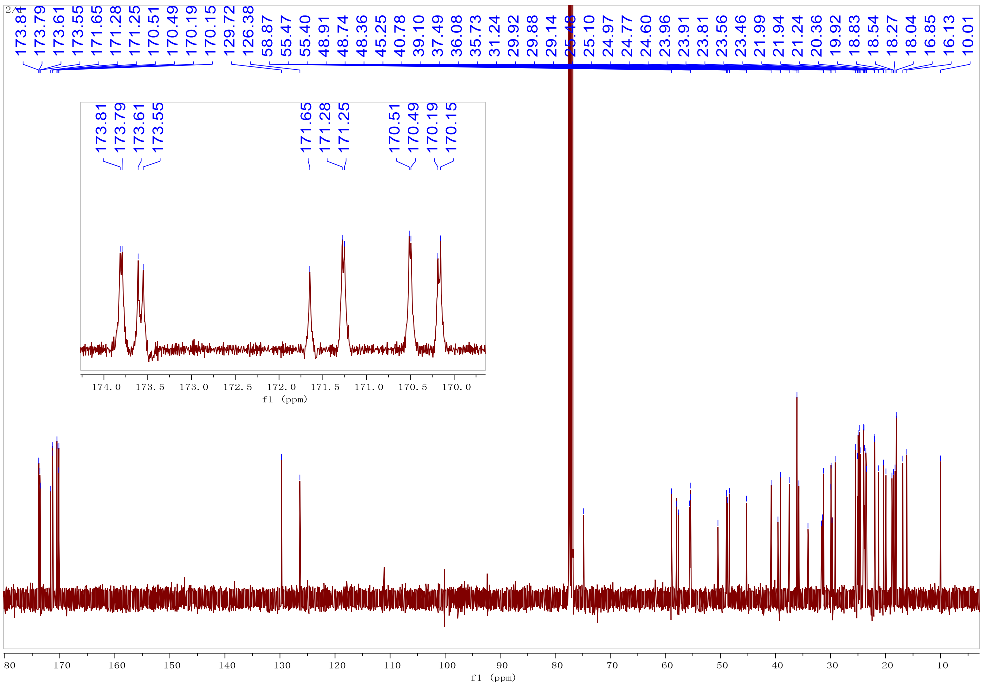

Figure S2. ^{13}C NMR of cyclosporin A (CsA).

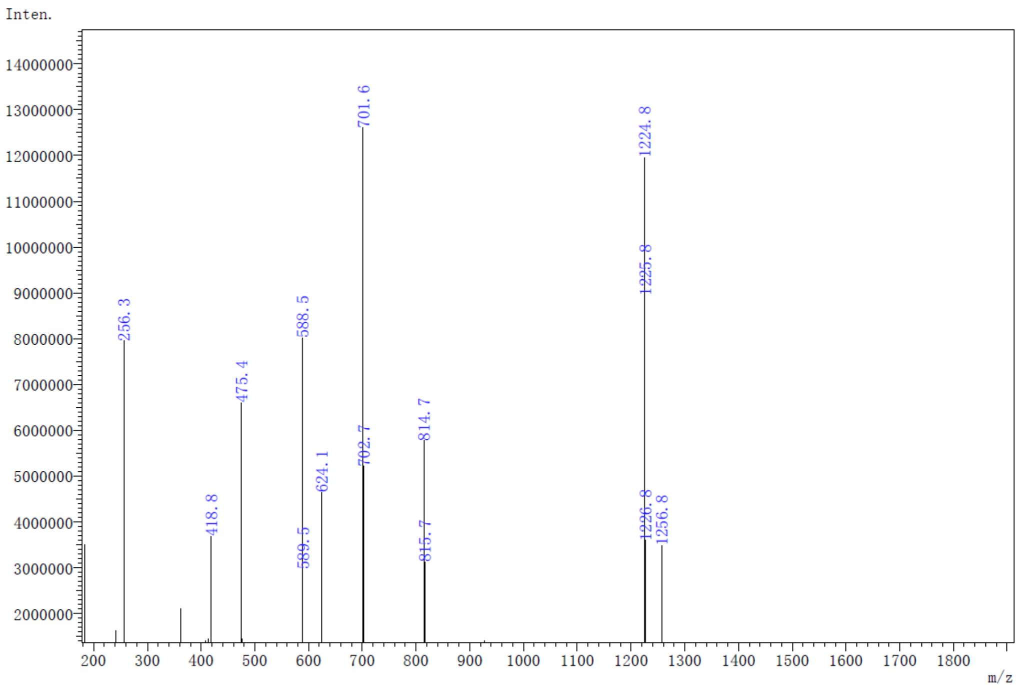

Figure S3. (+) ESI-MS of cyclosporin A (CsA).

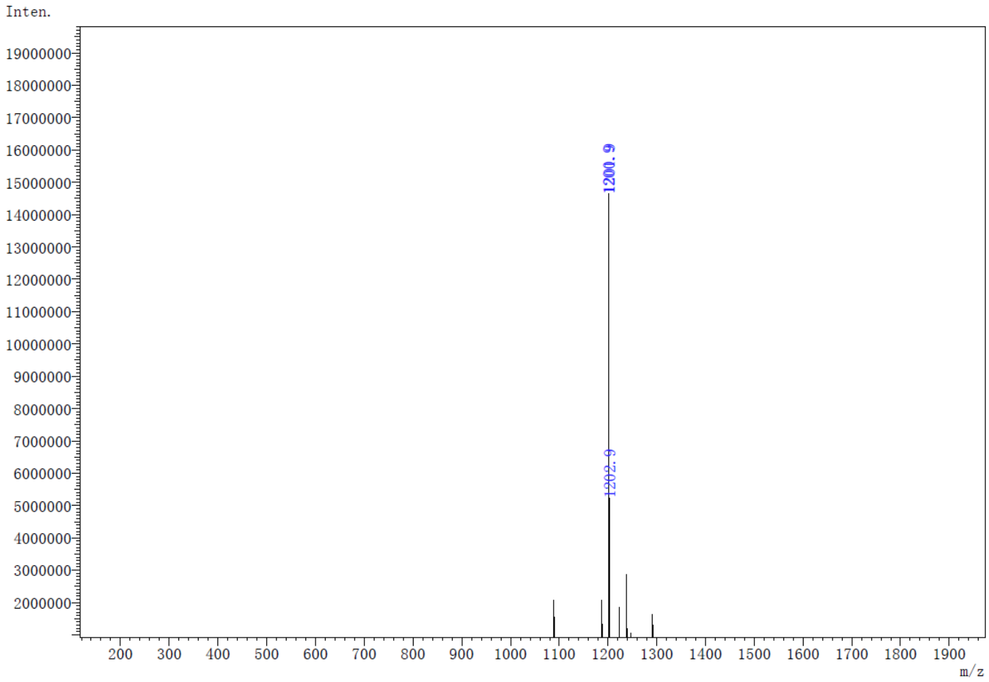

Figure S4. (−)ESI-MS of cyclosporin A (CsA).

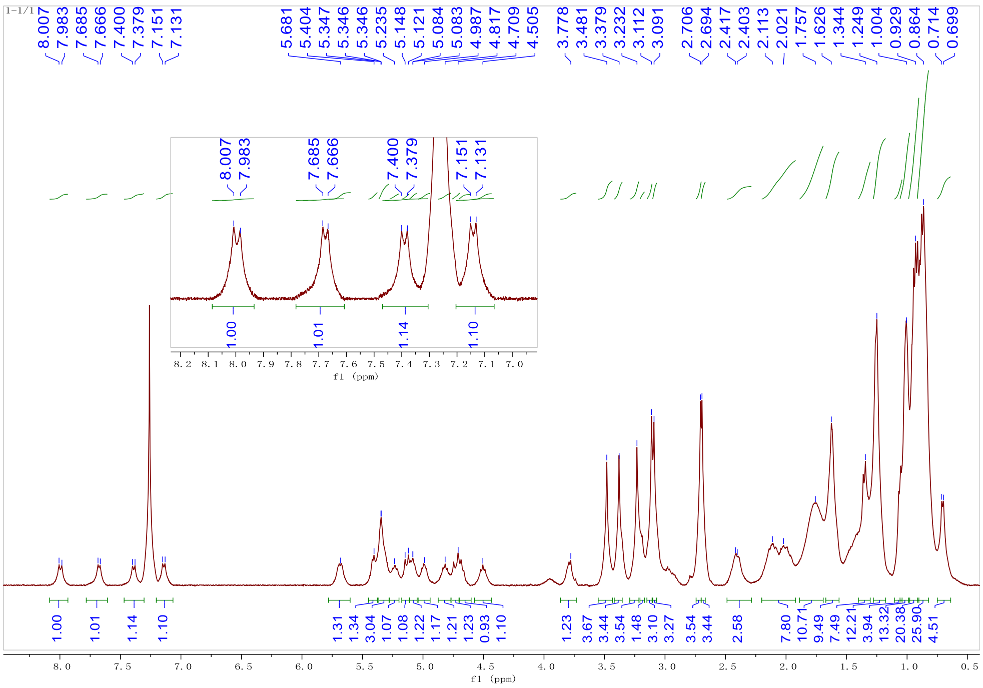

Figure S5. ^{1}H NMR of cyclosporin A (CsB).

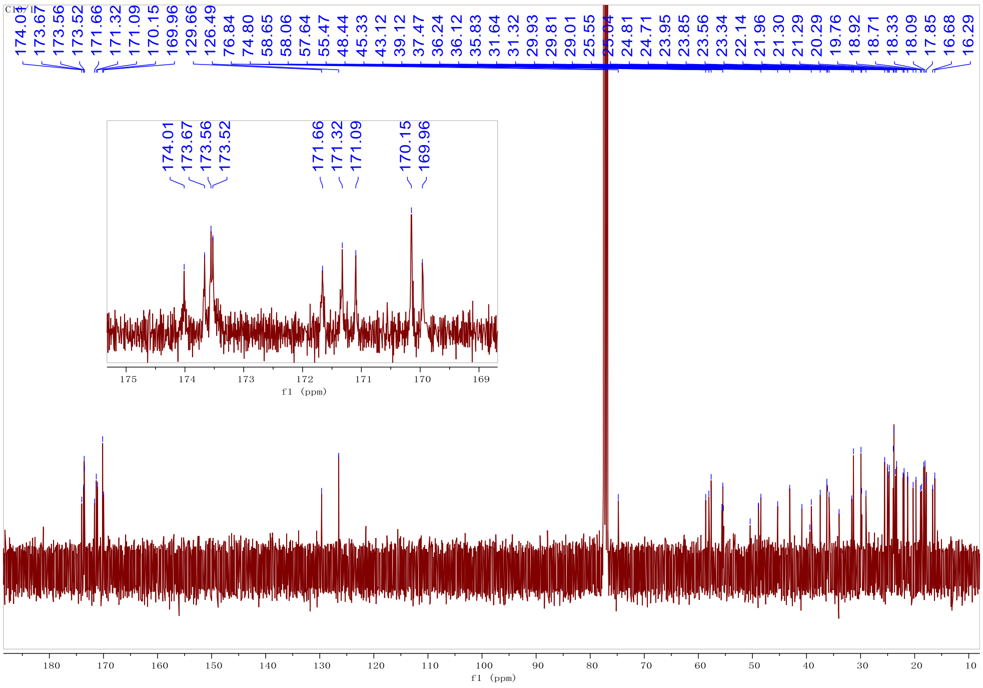

Figure S6. ^{13}C NMR of cyclosporin A (CsB).

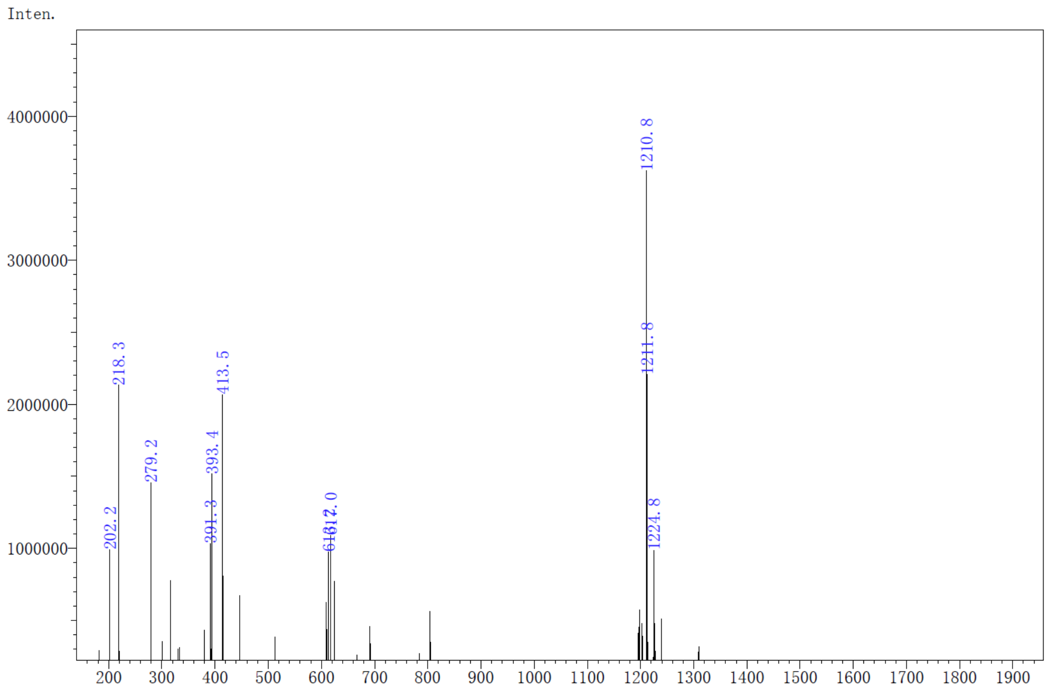

Figure S7. (+) ESI-MS of cyclosporin A (CsB).

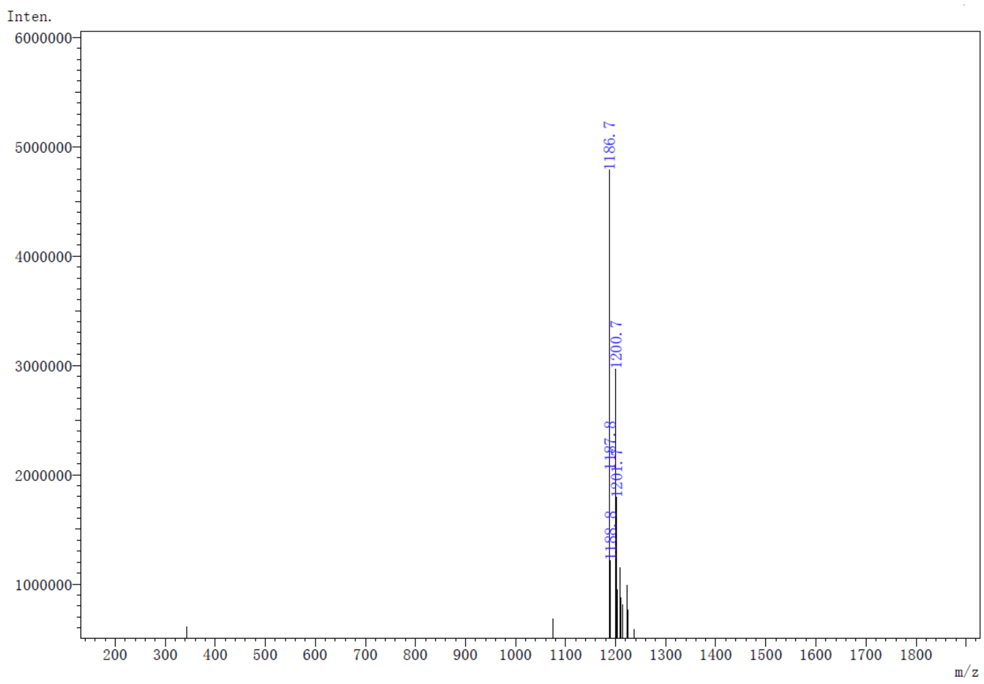

Figure S8. (−)ESI-MS of cyclosporin A (CsB).

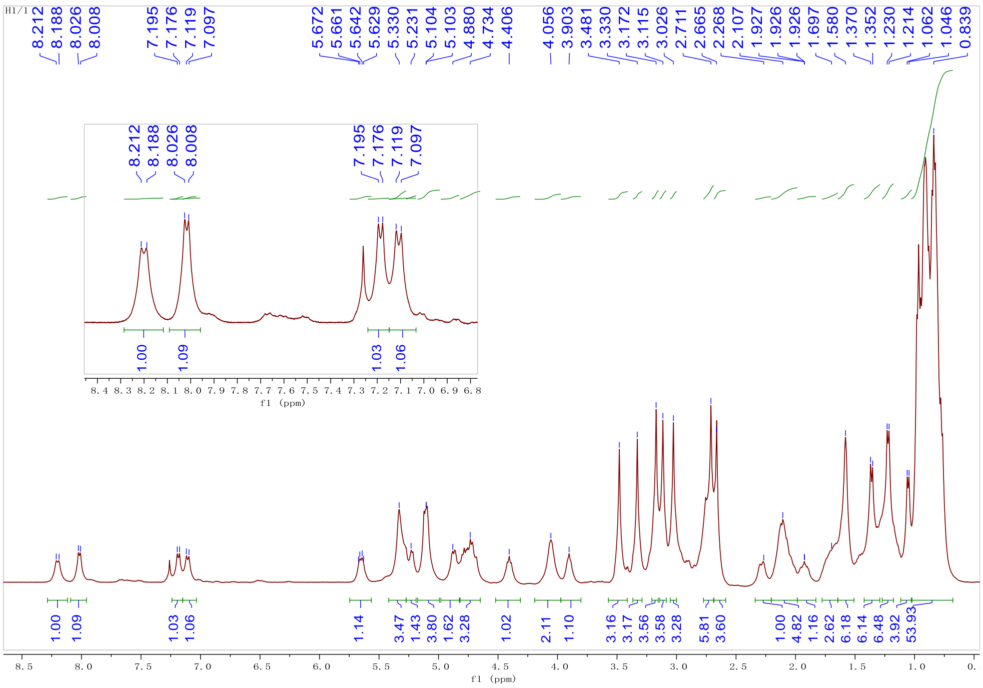

Figure S9. ^{1}H NMR of cyclosporin A (CsC).

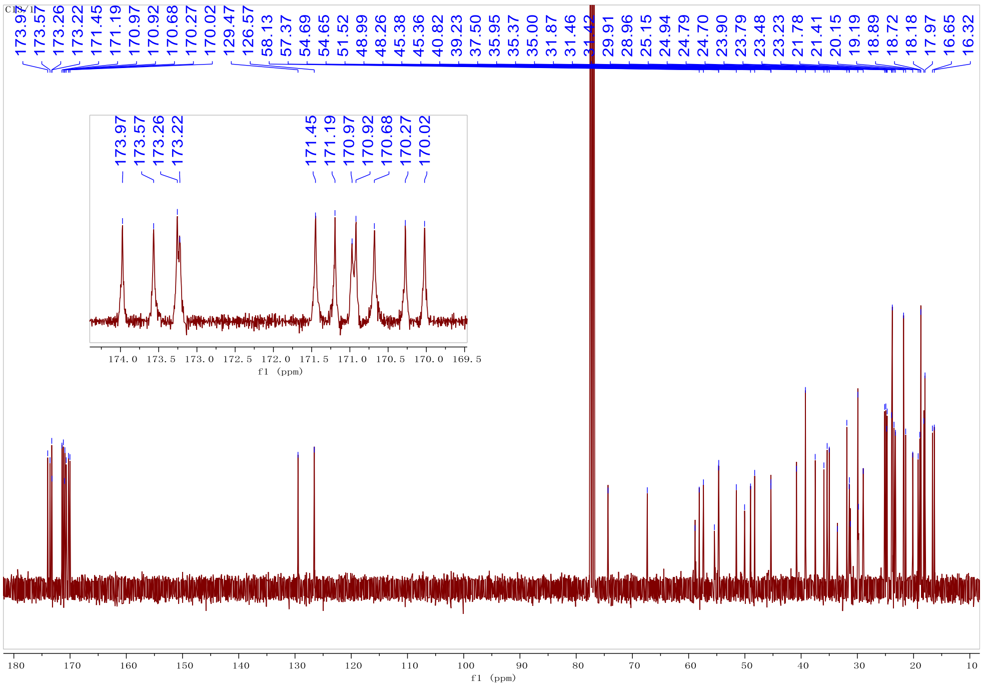

Figure S10. ^{13}C NMR of cyclosporin A (CsC).

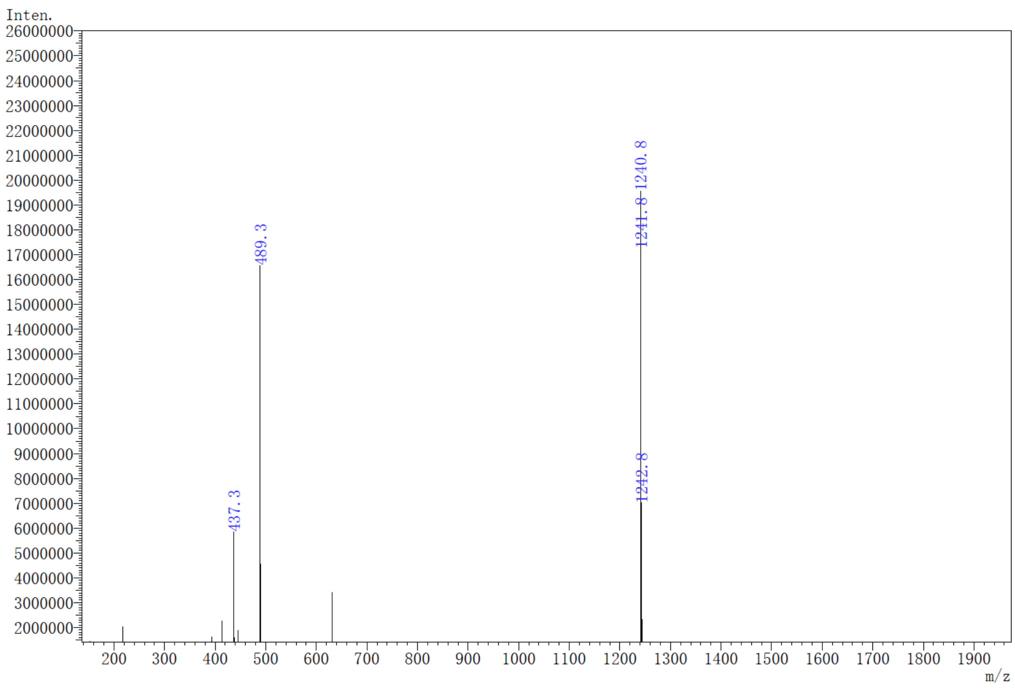

Figure S11. (+) ESI-MS of cyclosporin A (CsC).

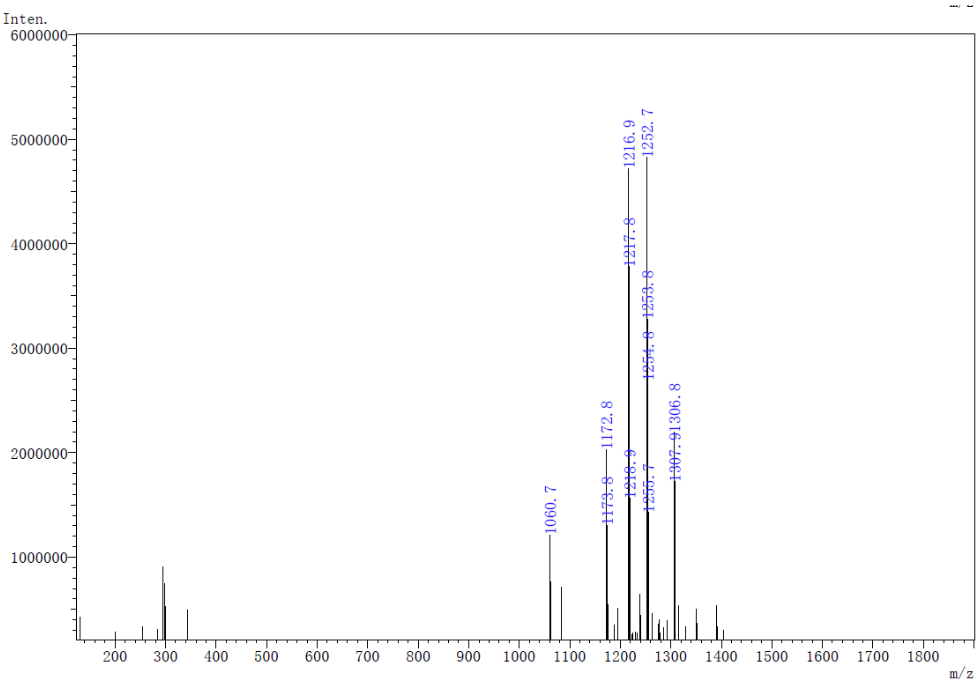

Figure S12. (−)ESI-MS of cyclosporin A (CsC).
